# Supplementary material for: Effects of plant tissue permeability on invasion and population bottlenecks of a phytopathogen
Source: Nat Commun. 2024 Jan 2;15:62. doi: 10.1038/s41467-023-44234-7 (PMC10762237; doi:10.1038/s41467-023-44234-7)
Supplement: Supplementary file 1 — Supplementary Information [file 41467_2023_44234_MOESM1_ESM.pdf]

Supplementary Information

Effects of plant-tissue permeability on invasion and population bottlenecks of a phytopathogen

Gaofei Jiang, Yuling Zhang, Min Chen, Josep Ramoneda, Liangliang Han, Yu Shi, Rémi Peyraud, Yikui Wang, Xiaojun Shi, Xinping Chen, Wei Ding, Alexandre Jousset, Yasufumi Hikichi, Kouhei Ohnishi, Fang-Jie Zhao, Yangchun Xu, Qirong Shen, Francisco Dini-Andreote, Yong Zhang, Zhong Wei

Supplementary Figures

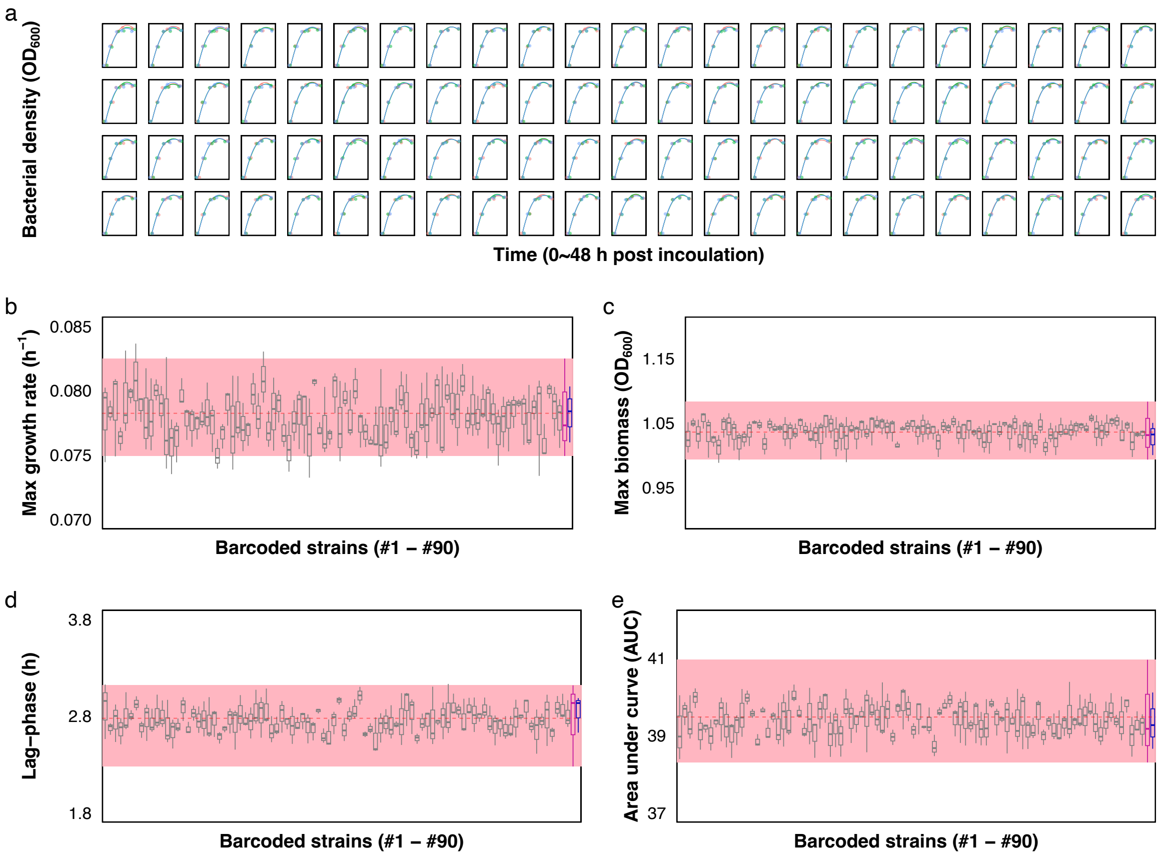

Supplementary Fig. 1. Evaluation of the potential effect of each of the 90 DNA barcodes on the strain fitness and virulence of *Ralstonia solanacearum* cultured in minimal medium. (**a**) Growth curves of the 90 barcoded strains compared to the wild-type strain (bottom left) and mixed 90 barcode pool populations (bottom right). Differences in maximum growth rate (**b**), biomass (**c**) and lag phase (**d**) and area under growth curve (**e**) of barcoded strains, wild-type strain (purple) and 90-barcode pool (blue). Each treatment was repeated three times (n = 3). The boxes represent the interquartile range of the 25 - 75th percentile of data, and lines represent medians. The purple and blue boxplots denote the ancestral wild-type strain and the equal mixture pool of the 90-barcoded strains in panels c and f, respectively.

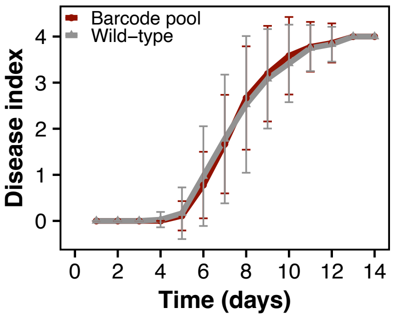

Supplementary Fig. 2. Evaluation of disease progress in a wilt-susceptible tomato cultivar (Ailsa Craig) inoculated with the wild-type strain and the pool of 90 barcoded *Ralstonia solanacearum*. Lines depict the standard deviation of the mean values.

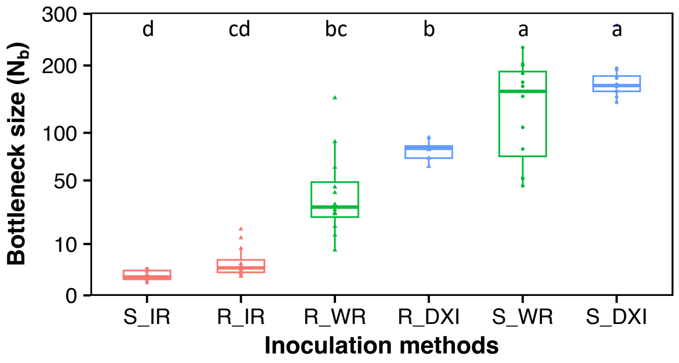

Supplementary Fig. 3. Differences in population bottlenecks of *Ralstonia solanacearum* across treatments. Comparison of bottleneck sizes (N_b) in *R. solanacearum* populations undergoing invasion in wilt-resistant (R) and wilt-susceptible (S) tomato cultivars based on inoculation methods (treatments). The treatments represent distinct plant–root permeability to invasion (i.e., intact roots, IR; wounded roots, WR; direct xylem inoculation, DXI). Each treatment was repeated 12 times (n = 12). The boxes represent the interquartile range of the 25th - 75th percentile of data, lines and dots represent medians and individuals.. Lowercase letters above box plots represent significant differences (one-way ANOVA with LSD test).

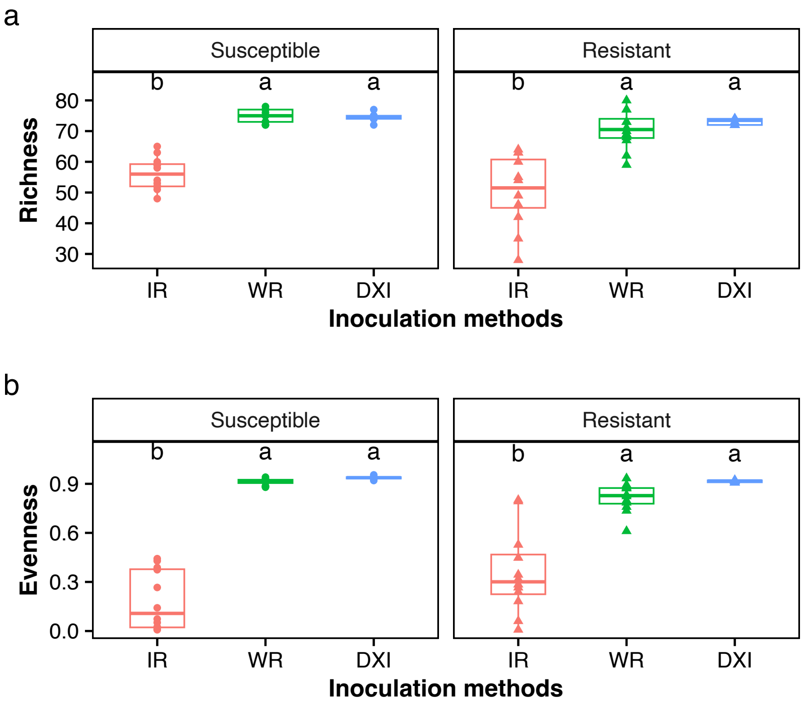

Supplementary Fig. 4. Comparison of the diversity of *Ralstonia solanacearum* populations colonizing susceptible and resistant plants across treatments. Diversity included observed richness (**a**) and Pielou's evenness (**b**). The treatments represent distinct plant–root permeability to invasion (i.e., intact roots, IR; wounded roots, WR; direct xylem inoculation, DXI). Each treatment was repeated 12 times (n = 12). The boxes represent the interquartile range of the 25th - 75th percentile of data, lines and dots represent medians and individuals. Lowercase letters above box plots represent significant differences (one-way ANOVA with LSD test).

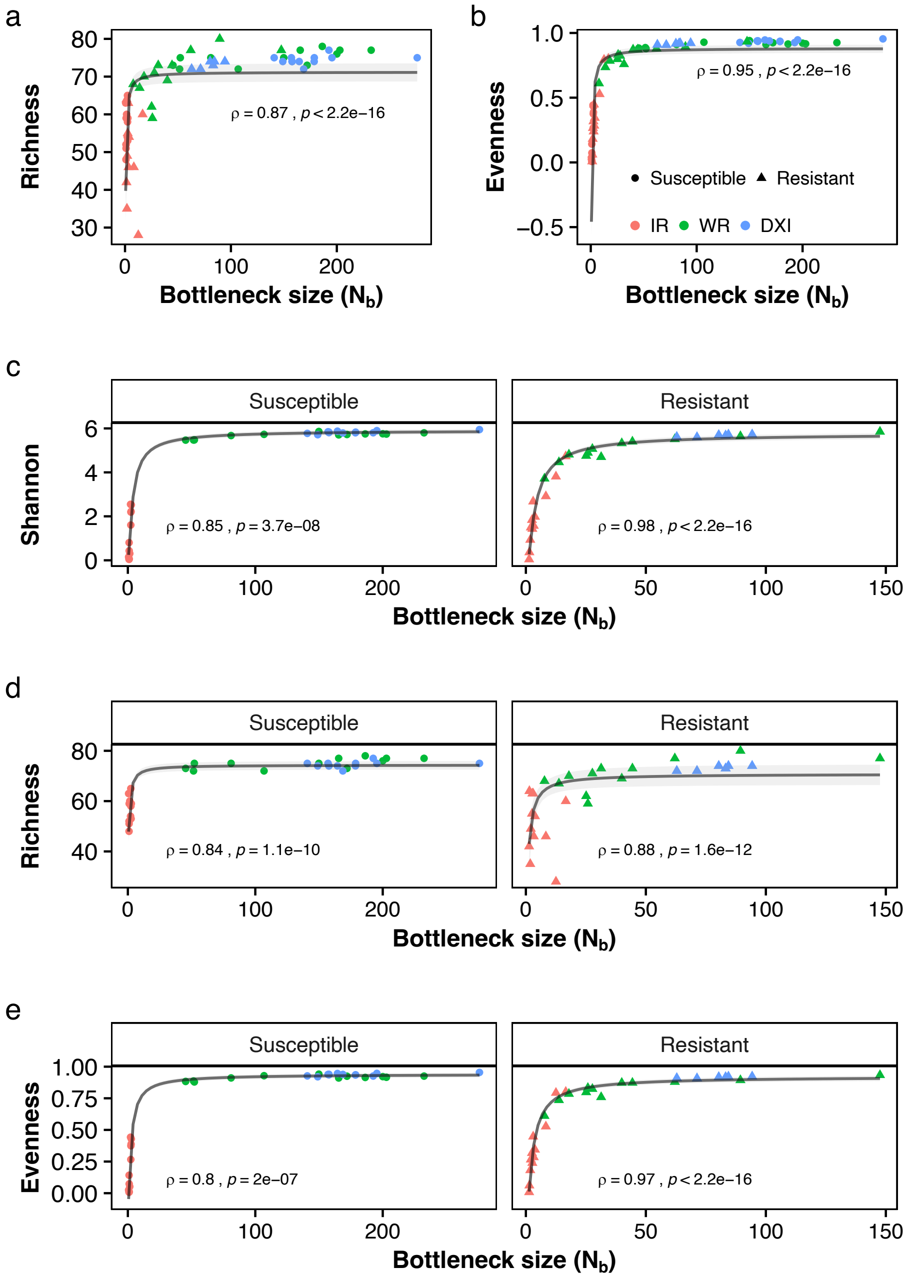

Supplementary Fig. 5. Correlation between bottleneck size (N_b) and the diversity of invasive *R. solanacearum* populations. The N_b values show a strong correlation with the observed richness (**a**) and Pielou's evenness (**b**) of *R. solanacearum* populations. This partten is not affected by the host genotype (susceptible vs resistant), as the diversity of invader populations is highly associated with N_b values. Diversity included the Shannon index (**c**), observed richness (**d**), and Pielou's evenness (**e**). Each treatment was repeated 12 times (n = 12). The treatments represent distinct plant–root permeability to invasion (i.e., intact roots, IR; wounded roots, WR; direct xylem inoculation, DXI). *Rho* (ρ) represents Spearman's rank correlation coefficient; Line and intersection represent the mean and 95% CI of the fitting curve of the asymptotic regression model.

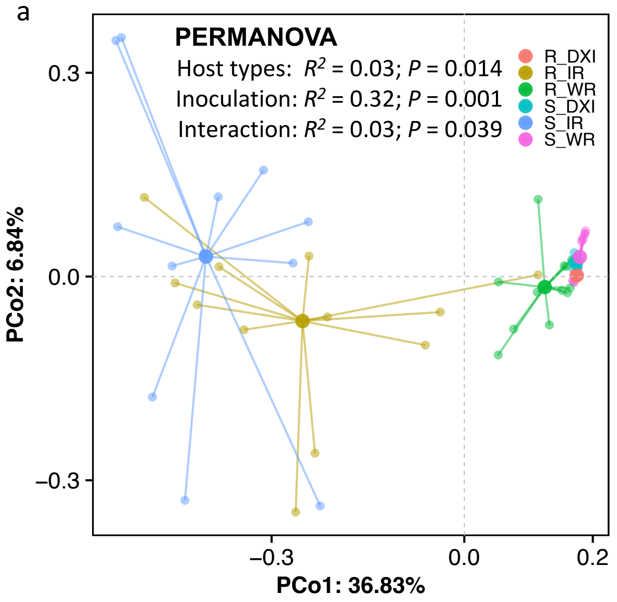

b

	PERMANOVA		Betadisper	
	R^2	*P* value	Diff in centroid	*P* value
**Overall**	0.38	**0.001**		**0.001**
**S_WR vs S_DXI**	0.26	**0.001**	-0.46	**0.000**
**S_WR vs R_WR**	0.15	**0.001**	0.01	0.993
**S_WR vs R_IR**	0.27	**0.001**	-0.13	**0.000**
**S_WR vs R_DXI**	0.28	**0.002**	-0.46	**0.000**
**S_IR vs S_WR**	0.38	**0.001**	0.05	0.114
**S_IR vs S_DXI**	0.37	**0.001**	0.47	**0.000**
**S_IR vs R_WR**	0.32	**0.001**	0.34	**0.000**
**S_IR vs R_IR**	0.08	**0.008**	0.00	1.000
**S_IR vs R_DXI**	0.38	**0.001**	0.51	**0.000**
**S_DXI vs R_WR**	0.19	**0.001**	-0.14	**0.000**
**S_DXI vs R_IR**	0.27	**0.001**	-0.47	**0.000**
**S_DXI vs R_DXI**	0.56	**0.001**	0.04	0.341
**R_WR vs R_DXI**	0.10	**0.001**	-0.34	**0.000**
**R_IR vs R_WR**	0.19	**0.001**	0.18	**0.000**
**R_IR vs R_DXI**	0.26	**0.001**	0.51	**0.000**

Community differences

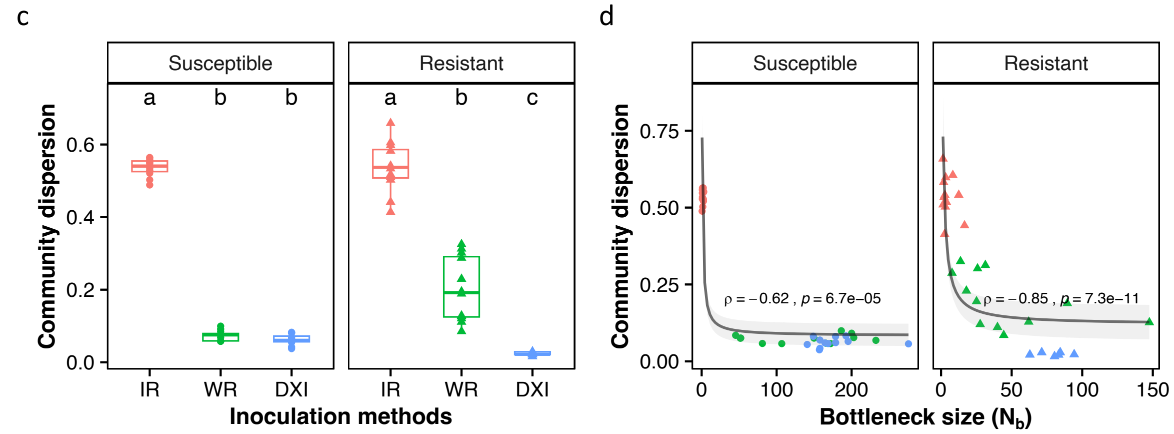

Supplementary Fig. 6. Compositional variation (a) of *Ralstonia solanacearum* populations colonizing susceptible (S) and resistant (R) tomato cultivars across treatments. PERMANOVA and betadisper (**b**) were used to test for differences in the invading pathogen populations and their dispersions. Panels (**c**) and (**d**) represent the differences in the dispersion of *R. solanacearum* lineage populations and their association with the population bottleneck size (N_b). The treatments represent distinct plant–root permeability to invasion (i.e., intact roots, IR; wounded roots, WR; direct xylem inoculation, DXI). S: susceptible and R: resistant. Lines and dots represent medians and individuals in panel (**c**).

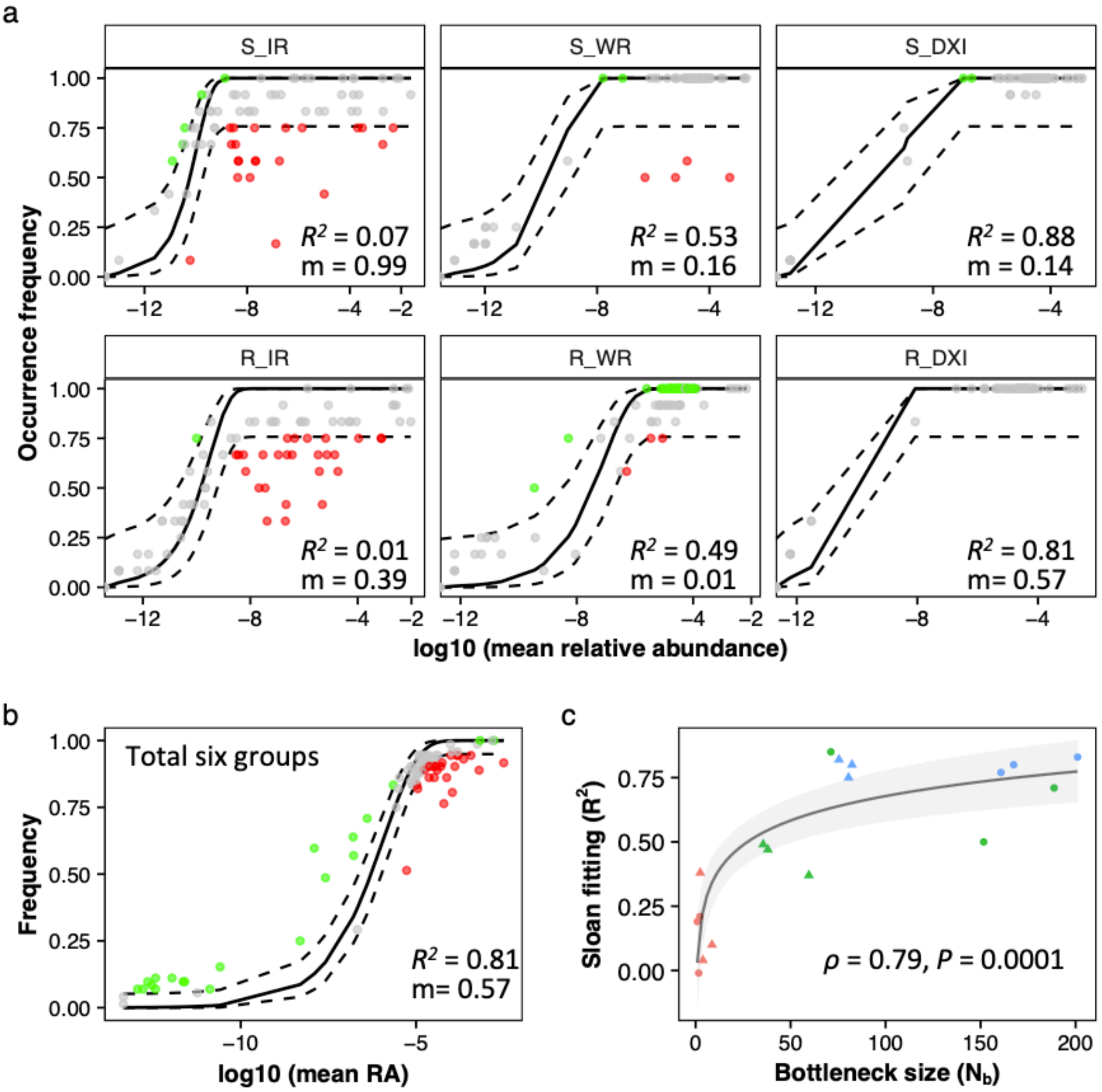

Supplementary Fig. 7. Sloan model distribution of the invading *Ralstonia solanacearum* populations. Frequency and abundance of barcoded strains invading susceptible (S) and resistant (R) tomato cultivars across treatments (**a**) and total samples (**b**). The treatments represent distinct plant–root permeability to invasion (i.e., intact roots, IR; wounded roots, WR; direct xylem inoculation, DXI). R^2: Goodness of fit of Sloan's model. m: rate of immigration (m) into the plant roots. Dashed lines represent 95% confidence intervals around the null model prediction. Barcoded strains that occurred more and less frequently than predicted by the model are displayed in red and blue, respectively. Panel (**c**) shows the correlation between the mean bottleneck size (N_b) and the goodness of fit values of the model.
