# Supplementary material for: Effects of plant tissue permeability on invasion and population bottlenecks of a phytopathogen
Source: Nat Commun. 2024 Jan 2;15:62. doi: 10.1038/s41467-023-44234-7 (PMC10762237; doi:10.1038/s41467-023-44234-7)
Supplement: Supplementary file 3 — Description of Additional Supplementary Files [file 41467_2023_44234_MOESM3_ESM.pdf]

Title: Supplementary Data 1.
Description: Description of the 25-bp DNA barcodes inserted into the chromosome of *Ralstonia solanacearum*.

Title: Supplementary Data 2.
Description: Frequencies at the start and end of the experiment of the barcoded *Ralstonia solanacearum* populations during invasion.
