# Supplementary material for: Effects of plant tissue permeability on invasion and population bottlenecks of a phytopathogen
Source: Nat Commun. 2024 Jan 2;15:62. doi: 10.1038/s41467-023-44234-7 (PMC10762237; doi:10.1038/s41467-023-44234-7)
Supplement: Supplementary file 6 — Reporting Summary [file 41467_2023_44234_MOESM6_ESM.pdf]

nature portfolio

Corresponding author(s): ZW & YZ

Last updated by author(s): Nov 20, 2023

Reporting Summary

Nature Portfolio wishes to improve the reproducibility of the work that we publish. This form provides structure for consistency and transparency in reporting. For further information on Nature Portfolio policies, see our Editorial Policies and the Editorial Policy Checklist.

Statistics

For all statistical analyses, confirm that the following items are present in the figure legend, table legend, main text, or Methods section.

n/a	Confirmed	
☐	☒	The exact sample size (n) for each experimental group/condition, given as a discrete number and unit of measurement
☐	☒	A statement on whether measurements were taken from distinct samples or whether the same sample was measured repeatedly
☐	☒	The statistical test(s) used AND whether they are one- or two-sided<br>*Only common tests should be described solely by name; describe more complex techniques in the Methods section.*
☐	☒	A description of all covariates tested
☐	☒	A description of any assumptions or corrections, such as tests of normality and adjustment for multiple comparisons
☐	☒	A full description of the statistical parameters including central tendency (e.g. means) or other basic estimates (e.g. regression coefficient) AND variation (e.g. standard deviation) or associated estimates of uncertainty (e.g. confidence intervals)
☐	☒	For null hypothesis testing, the test statistic (e.g. F, t, r) with confidence intervals, effect sizes, degrees of freedom and P value noted<br>*Give P values as exact values whenever suitable.*
☒	☐	For Bayesian analysis, information on the choice of priors and Markov chain Monte Carlo settings
☐	☒	For hierarchical and complex designs, identification of the appropriate level for tests and full reporting of outcomes
☐	☒	Estimates of effect sizes (e.g. Cohen's d, Pearson's r), indicating how they were calculated

Our web collection on statistics for biologists contains articles on many of the points above.

Software and code

Policy information about availability of computer code

Data collection	The data were collected by experiments and sequencing.
Data analysis	Statistical analyses were performed in R v4.1.1 63. We used the vegan package v2.6-2 64 to estimate alpha diversity indices (i.e., observed richness, Shannon index, and Pielou evenness). Analysis of variance (ANOVA) was used to determine the statistical significance of cultivars and infection treatments and their interaction effect using the package agricolae v1.3-5 65. Principal coordinate analysis (PCoA) based on Bray–Curtis distances was carried out using the pcoa function in the ape package v5.6-2 66. Permutational multivariate analysis of variance (PERMANOVA) was used to test for significant differences in inoculation treatments using the adonis function in the vegan package v2.6-2 64. Permutation multivariate analysis of dispersion (PERMDISP) was used to test for differences in multivariate dispersion of R. solanacearum barcoded strains within each inoculation treatment using the betadisper function in the vegan package v2.6-2 64. Correlations between diversity indices and the R. solanacearum dispersion axes to Nb values were performed using Spearman's rank correlations implemented in the cor.test function of ggpubr package v0.4.0 67. Curves were fitted with an asymptotic regression model (concave for the diversity indices and convex for dispersion) in response to the exponential of reciprocal Nb values using the lm function in the stats package v4.1.1 63. The Nb breakpoint (Nbp) of fitting curves was fixed using the segmented function in the segmented package v1.6-4 68.

For manuscripts utilizing custom algorithms or software that are central to the research but not yet described in published literature, software must be made available to editors and reviewers. We strongly encourage code deposition in a community repository (e.g. GitHub). See the Nature Portfolio guidelines for submitting code & software for further information.

Data

Policy information about availability of data

All manuscripts must include a data availability statement. This statement should provide the following information, where applicable:

- Accession codes, unique identifiers, or web links for publicly available datasets
- A description of any restrictions on data availability
- For clinical datasets or third party data, please ensure that the statement adheres to our policy

All data underlying this study are available in RsBarcode [https://doi.org/10.5281/zenodo.7807998] at Zenodo, and all raw sequencing data are deposited at the National Genomics Data Center of China (ID: PRJCA016125 [https://ngdc.cncb.ac.cn/bioproject/browse/PRJCA016125]). Source data are provided with this paper.

Research involving human participants, their data, or biological material

Policy information about studies with human participants or human data. See also policy information about sex, gender (identity/presentation), and sexual orientation and race, ethnicity and racism.

Reporting on sex and gender	N/A
Reporting on race, ethnicity, or other socially relevant groupings	N/A
Population characteristics	N/A
Recruitment	N/A
Ethics oversight	N/A

Note that full information on the approval of the study protocol must also be provided in the manuscript.

Field-specific reporting

Please select the one below that is the best fit for your research. If you are not sure, read the appropriate sections before making your selection.

☐ Life sciences ☐ Behavioural & social sciences ☒ Ecological, evolutionary & environmental sciences

For a reference copy of the document with all sections, see nature.com/documents/nr-reporting-summary-flat.pdf

Ecological, evolutionary & environmental sciences study design

All studies must disclose on these points even when the disclosure is negative.

Study description	We designed a full factorial experiment using a wilt-susceptible (Ailsa Craig) and a wilt-resistant (Hawaii 7996) tomato cultivar. This experimental design (2 cultivars x 3 invasion methods x 12 replicates) was used to evaluate the extent to which decreasing levels of permeability reflect reduced magnitudes of the selective filters to pathogen invasion (no barrier removal, IR; external epidermis barrier removal by artificial root wounds, WR; and total barrier removal via direct xylem inoculation, DXI)
Research sample	The treatments are associated with the permeability of the plant's physical barriers to phytopathogen invasion (intact roots, IR; wounded roots, WR; direct xylem inoculation, DXI); Wilt-susceptible (Ailsa Craig) and a wilt-resistant (Hawaii 7996) tomato cultivar.
Sampling strategy	Each treatment included 12 repeats (n = 12).
Data collection	2021
Timing and spatial scale	May 2021 - Dec 2021
Data exclusions	No data are excluded.
Reproducibility	Experimental findings are reproducible.
Randomization	data collected were randomized.
Blinding	N/A

Did the study involve field work? ☐ Yes ☒ No

Reporting for specific materials, systems and methods

We require information from authors about some types of materials, experimental systems and methods used in many studies. Here, indicate whether each material, system or method listed is relevant to your study. If you are not sure if a list item applies to your research, read the appropriate section before selecting a response.

Materials & experimental systems

n/a	Involved in the study	
☒	☐	Antibodies
☒	☐	Eukaryotic cell lines
☒	☐	Palaeontology and archaeology
☒	☐	Animals and other organisms
☒	☐	Clinical data
☒	☐	Dual use research of concern
☐	☒	Plants

Methods

n/a	Involved in the study	
☒	☐	ChIP-seq
☒	☐	Flow cytometry
☒	☐	MRI-based neuroimaging

Dual use research of concern

Policy information about dual use research of concern

Hazards

Could the accidental, deliberate or reckless misuse of agents or technologies generated in the work, or the application of information presented in the manuscript, pose a threat to:

No	Yes	
☒	☐	Public health
☒	☐	National security
☐	☒	Crops and/or livestock
☐	☒	Ecosystems
☒	☐	Any other significant area

Hazards: N/A

For examples of agents subject to oversight, see the United States GovernmentPolicy for Institutional Oversight of Life Sciences Dual Use Research of Concern .

Experiments of concern

Does the work involve any of these experiments of concern:

No	Yes	
☒	☐	Demonstrate how to render a vaccine ineffective
☒	☐	Confer resistance to therapeutically useful antibiotics or antiviral agents
☒	☐	Enhance the virulence of a pathogen or render a nonpathogen virulent
☒	☐	Increase transmissibility of a pathogen
☒	☐	Alter the host range of a pathogen
☒	☐	Enable evasion of diagnostic/detection modalities
☒	☐	Enable the weaponization of a biological agent or toxin
☒	☐	Any other potentially harmful combination of experiments and agents

Precautions and benefits

Biosecurity precautions	N/A
Biosecurity oversight	N/A
Benefits	N/A
Communication benefits	N/A

Plants

Seed stocks	N/A
Novel plant genotypes	N/A
Authentication	N/A
